# Assessing the impact of different donor milk treatments on infant health and growth: a systematic review protocol

**DOI:** 10.1136/bmjopen-2024-087653

**Published:** 2024-12-27

**Authors:** Daniel Klotz, Agnieszka Bzikowska-Jura, Marzia Giribaldi, Tanya Cassidy, Laura Cavallarin, Serena Gandino, Karolina Karcz, Chiara Peila, Carolyn Smith, Bartłomiej Walczak, Aleksandra Wesolowska

**Affiliations:** 1 Bethel Center for Pediatrics, Department of Neonatology and Pediatric Intensive Care, University Hospital OWL, University of Bielefeld, Bielefeld, Germany; 2 Laboratory of Human Milk and Lactation Research, Department of Medical Biology, Medical University of Warsaw, Warsaw, Poland; 3 Institute of Sciences of Food Productions, National Research Council, Grugliasco, Torino, Italy; 4 Kathleen Lonsdale Institute for Human Health Research, Maynooth University, Maynooth, Ireland; 5 Nuffield Department of Women's & Reproductive Health, University of Oxford, John Radcliffe Hospital, Oxford, UK; 6 Department and Clinic of Neonatology, Wroclaw Medical University, Wroclaw, Poland; 7 Neonatal Unit, Department of Public Health and Pediatrics, University of Turin, Torino, Italy; 8 Bodleian Health Care Libraries, University of Oxford, Banbury, UK; 9 Institute of Applied Social Sciences, University of Warsaw, Warszawa, Poland

**Keywords:** NEONATOLOGY, Meta-Analysis, Neonatal intensive & critical care, NUTRITION & DIETETICS

## Abstract

**ABSTRACT:**

**Introduction:**

Donor human milk (DHM) is the first alternative if mother’s own milk is unavailable or contraindicated. Much DHM research has focused on its nutritional, immunological and biochemical composition in response to various maternal variables, standard human milk banking procedures and storage protocols. The current systematic review protocol, however, aims to systematically gather and analyse existing data pertaining to the impact of these aforementioned factors on the clinical, health-related and developmental outcomes observed in infants fed with DHM.

**Methods and analysis:**

We will apply a predefined search strategy including Cochrane Central Register of Controlled Trials, Global Index Medicus, CINAHL on Ebscohost, Medline, Embase, Emcare, Pubmed, Global Health on OVID, Google Scholar, clinicaltrials.gov, the WHO International Trials Registry and Platform and the European Union/European Economic Area Clinical Trials Register. No setting, patient population, date or language restrictions will be applied. The search strategy will consist of search words on the key concepts of ‘donated human milk’ and ‘effect on infants’. Published or unpublished primary research studies are eligible for inclusion if there is an abstract available in English. Conference proceedings, animal studies and publications with no original data will be excluded.

Authors of unpublished or partially published studies will be contacted and eligible data added if provided. Risk of bias will be evaluated according to CASP using appropriate tools depending on study type (RoB 2, ROBINS-I, Newcastle Ottawa Scale). Data will be synthesised in a quantitative format describing significant results as well as presenting the results of the quality assessment of studies.

**Ethics and dissemination:**

This systematic review does not require ethical approval, as we will not collect primary data. The review will be published in a peer-reviewed journal and disseminated electronically and in print.

**PROSPERO registration number:**

CRD42024522015.

**STRENGTHS AND LIMITATIONS OF THIS STUDY**

- This review will be the first systematic approach to characterise the influence of a wide range of donor variables and human milk banking practices range on the clinical, health-related and developmental outcomes observed in infants fed with donor human milk.
- This protocol adheres to the Preferred Reporting Items for Systematic Reviews and Meta-Analyses 2020 reporting guideline and will be applied by an experienced and diverse team in human milk science.
- One potential methodological constraint involves the exclusion of publications lacking an English abstract; this limitation could be mitigated by the absence of restrictions regarding time, language or setting.

## Introduction

 Breastfeeding has been recognised as the gold standard for infant nutrition. Whenever mother’s own milk (MOM), either at the breast or expressed, is unavailable or contraindicated, the next best option, in particular for vulnerable preterm and low birth weight infants, is represented by donor human milk (DHM).^1^

Feeding DHM, either as the sole dietary source or as a supplement to MOM, reduces the incidence of necrotising enterocolitis, when compared with formula.^2 3^ Observations of benefits were also noted, although supported by evidence of lesser strength, correlating with enhancements in feeding tolerance, expedited attainment of full feeds and reduced duration of hospitalisation.^4^ The purported inferiority of DHM in meeting targeted growth milestones in comparison to formula feeding has been revisited recently and appears to lack substantial support from the latest research findings.^5^

Despite recommendations for DHM and the global expansion of human milk banks (HMBs), little authoritative guidance exists on the implementation, operation and regulation of HMBs.^6^ In 2019, PATH made available a toolkit for establishing and integrating HMB programmes,^7^ and, more recently, the WHO initiated a process of developing standards on DHM banking.^8^ Thus far, a significant portion of existing research data has concentrated on elucidating and documenting the nutritional, immunological and biochemical composition of DHM in response to various maternal variables, such as lactation progression and birth outcomes (ie, premature vs term delivery), as well as standard HMB procedures including pasteurisation and storage protocols.

One of the crucial steps needed to implement HMB worldwide is ‘to establish global standards on minimum requirements of safety and quality with guidance addressing all aspects of human milk banking (screening, donation, storage, pooling, pasteurisation and processing, microbial screening and allocation of DHM)’.^6^ These standards should be established based on scientific evidence regarding the benefits or disadvantages of specific factors and practices and on the actual clinical outcomes observed in infants receiving DHM, in conjunction with the available indications obtained on milk composition and contaminants.

Milk donations undergo several processing steps, ranging from donor selection to milk collection using various techniques/equipment in diverse settings, followed by refrigerated/frozen storage for varying durations, pasteurisation employing different methods or provision as raw milk, with appropriate testing before and/or after pasteurisation. It is anticipated that each of these steps may impact the composition and safety of DHM to varying degrees. Previous reviews have addressed some aspects of the influence of these steps on milk composition and safety.^4 9^

The current systematic review protocol aims to systematically gather and analyse existing data pertaining to the impact of the aforementioned factors on the clinical, health-related and developmental outcomes observed in infants fed with DHM. In clinical practice, the majority of DHM is prioritised for very low birthweight or critically ill infants, including infants with conditions like congenital heart disease, abdominal surgical issues, intrauterine growth restriction or small for gestational age. While less common, DHM might also be considered for late preterm and term infants who may require short-term support in the newborn nursery. Our review will include as participants all infants who have received DHM, exclusively or in conjunction with MOM or with formula, irrespective of the clinical indication and of the actual amount of DHM received.

## Methods and analysis

### Eligibility criteria

An adapted patient population, intervention/exposure, comparison, outcome and study design criteria format was used to guide the parameters of inclusion criteria (table 1). Published or unpublished primary research studies are eligible for inclusion. We will not impose any restriction on the language of the report, provided that the abstract is available in English. Translations of non-English manuscripts will be performed using large language models. We will not apply setting, patient population or time frame restrictions. We will exclude conference proceedings, animal studies and publications with no original data—including reviews, editorials, comments, letters, case studies and book chapters. However, the reference list of relevant reviews will be screened manually to identify potentially additional eligible studies.

**Table 1:** Adapted patient population, intervention/exposure, comparison, outcome and study design criteria for study inclusion*

Parameter	Inclusion criteria
Population	Are infants receiving DHM (exclusively or in conjunction with MOM or with formula and irrespective of the actual amount of DHM received)
Intervention 1	When donors are characterised by different lactation stages
Intervention 2	When donors are characterised by different birth outcomes (ie, premature vs delivery at term)
Intervention 3	When donors are characterised by different dietary regimens
Intervention 4	When donors are characterised by different lifestyle factors (eg, smoking, increased infectious or toxicological risks)
Intervention 5	When donors are characterised by different health status (eg, acute or chronic illness, BMI outside healthy limit)
Intervention 6	When donated milk is expressed with different methods
Intervention 7	When different hygiene practices or settings are used during donated milk expression
Intervention 8	When donated milk is pasteurised with different methods or fed raw (ie, unpasteurised)
Intervention 9	When different testing procedures are used on donations (ie, for microbial contamination)
Outcome	Differently affected in their growth, development and/or health status (any reported outcome)
Study design	Any

* according to WHO guidance.

BMIbody mass indexDHMdonor human milkMOMmother's own milk

If unpublished or partially published results might be eligible, the authors will contact the author/researcher/contact person by email, who will be asked for additional information to integrate additional data.

### Information sources and search strategy

Using an a priori research protocol (PROSPERO CRD42024522015), designed with a health sciences librarian, the search will include the following electronic databases: Cochrane Central Register of Controlled Trials (CENTRAL), Global Index Medicus, CINAHL on Ebscohost, Medline, Embase, Emcare, Pubmed, Global Health on OVID and Google Scholar.

We will search clinical trial registries for recently completed trials: CENTRAL, clinicaltrials.gov, the WHO International Trials Registry and Platform and the European Union and European Economic Area clinical trials register. No date restrictions will be applied.

We will apply a hybrid search strategy, by combining structured database searches with snowballing searches, in order to identify the highest number of relevant primary studies.^10^

Regarding the database searches, the search strategy will consist of search words on the key concepts of ‘donated human milk’ and ‘effect on infants’ (online supplemental file 1). These search terms will be adapted for use with the other bibliographic databases. The 10 most cited articles among those retrieved by database search and included in the systematic review will be manually searched for in PubMed and Google Scholar for reference lists, cited by and similar articles.^11^ The first 200 records for each of these articles found in this way in each database will undergo the same screening process as the parent article. Additionally, the reference list of relevant reviews will be screened manually to identify potentially additional relevant studies.

The searches are planned to be performed in April to June 2024.

### Data management

Our systematic review will follow the methodological recommendations of the Cochrane Collaboration,^11^ in accordance with WHO recommendations and PRISMA (Preferred Reporting Items for Systematic Reviews and Meta-Analyses) guidelines.^12^ The PRISMA for systematic review protocols (PRISMA-P) was used to prepare this protocol^13^ (online supplemental file 2). The raw data collected are publications containing no sensitive data and available in text formats (pdf, docx, rtf, etc). The papers selected for a full-text review will be stored in the COVIDENCE system (Veritas Health Innovation, Melbourne, Australia). The system will allow to track each step in the process, from uploading files to study selection, to data extraction and also allowing to resolve controversies arising along the process. Additional backups will be stored on the team’s shared drive and offline. The data extracted with the COVIDENCE (xls) and further transformed into sav format will be backed in the same way. The backups will be managed by one appointed team member.

### Study selection

The systematic literature search on selected databases will be conducted by the health science librarian. The search results from different databases will be merged using COVIDENCE and duplicates will be removed by the software. Each article will be independently screened by two authors with previous experience in the field of DHM banking. This screening stage will use titles and abstracts to remove irrelevant reports on the basis of eligibility and inclusion criteria. When uncertainty about inclusion of the study arises, the study paper will proceed to the full-text screening stage. Once the initial title and abstract screening is complete, all remaining studies will be retrieved and independently screened in full text by two authors. A third review author will be involved at the end of this stage to resolve conflicts.

### Data extraction

A standardised data extraction form will be used to report study characteristics, participants, interventions and outcomes, in COVIDENCE (online supplemental file 3). Two review authors will independently extract the data of interest from the included studies and evaluate the study’s quality using the online forms. The extracted data will be cross-checked, and, if any difference will be identified, the full review team will discuss the decision until it is resolved.

If study data are only available from figures, data will be extracted by use of the validated software PlotDigitizer (https://plotdigitizer.com). Missing or uncertain data will be handled by tentative contact with the study investigator by email for data or additional details. The same approach will be followed for ongoing studies and clinical trials. Data extracted with COVIDENCE will be transferred to sav. format for further statistical analysis with SPSS software (IBM SPSS Statistics for Windows, Armonk, NY, USA).

### Outcomes

Health and growth outcomes of infants fed donor milk are detailed in box 1. We aim to incorporate any reported variations of health and growth outcome parameters to ensure inclusivity across all relevant outcome variables. Due to possible differences in target clinical outcomes of interest for studies addressing different populations, the outlined search strategy includes general terms that might be suitable to collect studies addressing all possible clinical outcomes besides those listed as example in box 1. These include terms such as: ‘Outcome*’, ‘Consequenc*’, ‘Issue*’, ‘Effect*’, ‘Benefit*’, ‘Harm*’, ‘Risk factor*‘, ‘Complication*’ and ‘Illness*’. Therefore, box 1 could be further enriched with other outcomes when appropriate.

Box 1 **Health, growth and other outcomes**

**Health outcomes**

- Morbidity (including BPD, NEC, ROP, PDA, IVH)
- Mortality (any timepoint and cause)
- Feeding tolerance during hospital stay (defined by duration of parenteral nutrition, time to full enteral feeding or predefined feeding intolerance score)
- Adverse events (any unwanted medical occurrence in donor human milk receiving patients regardless of whether it is related to the intervention or not^19^
- Infections (any aetiology)
- Nutritional deficiencies (vitamins and/or micronutrients)
- Neurodevelopment (any neurodevelopmental assessment)

**Growth and development outcomes**

- any

**Other**

- Duration of parenteral feeding
- Length of hospital stay

BPD, bronchopulmonary dysplasia; IVH, intraventricular haemorrhage; NEC, necrotisiizing enterocolitis; ROP, retinopathy of prematurity; PDA, patent ductus arteriosus; ROP, retinopathy of prematurity.; IVH, intraventricular haemorrhage

### Risk of bias and quality assessment tools

Two reviewers will assess each included study for risk of bias (RoB), independently. In case of disagreement, the full review team will be asked for consultation. All eligible studies will be formally evaluated using standardised tools. CASP^14^ and RoB indicators will be included in the quality control form. For studies with randomised controlled trials, the RoB will be assessed by using RoB 2.^11^ For non-randomised controlled trials, the RoB in Non-Randomised Studies of Interventions (ROBINS-I) tool is used.^15^ For observational studies, the Newcastle Ottawa Scale will be considered.^16^

### Metabiases

We will conduct tests for statistical heterogeneity, as advocated in ref. 12. To assess statistical heterogeneity among the included studies, we will conduct a Cochran’s Q test using SPSS and create a forest plot representation of these data. The Cochran’s Q test evaluates whether the observed variation in the effect sizes across studies is greater than expected.

### Confidence in cumulative evidence

We will use the grades of recommendation, assessment, development and evaluation (GRADE) approach, as outlined in the GRADE handbook,^17^ to assess the certainty of evidence for clinically relevant outcomes. Two review authors will independently assess the certainty of the evidence for each of the outcomes of interest. In the case of a small sample size or small number of studies, we will consider the potential for publication bias and follow the GRADE guidelines specifically for rating quality of evidence–publication bias.^18^

### Data synthesis

Two methods of synthesis will be provided. A quantitative data synthesis will be grouping similar data and present the results (distribution, mean, SD and effect size when available) in tables and graphical presentation—forest plots, for example, for combining the results of multiple clinical trials to show point estimates arising from different studies concerning the impact of the expression method, lactation stage, heat treatment and specific clinical and lifestyle factor (for DHM) over the growth and health outcomes of the infants. On the forest plots, mean values with CIs for each study will be provided. Additionally, each mean will be plotted relative to the vertical line of no difference. An analysis will be performed using Excel and SPSS software.

Qualitative data synthesis as a thematic analysis will identify and code information about the selected studies’ methodologies and findings. Codes will be organised into subheadings and descriptive categories, developing these categories into analytical themes. An analysis of these themes will be performed using specialised software (MAXQDA, Verbi, Germany).

### Ethics and dissemination

This systematic review does not require ethical approval as primary data will not be collected. The primary audiences for dissemination of this study results include healthcare professionals, policy makers, community stakeholders and scientific community. In order to reach those audiences, the research findings will be used by the WHO Department of Nutrition and Food Safety to inform the guidance on DHM banking. The research findings, with permission from the WHO, will be submitted for publication in a reputable peer-reviewed open access interdisciplinary journal. The authors intend to publish the data supporting the findings of this study within the article and/or its supplementary materials. Additional data may be made available on reasonable request by the corresponding author.

### Patient and public involvement

The research question involved in this systematic review was provided by the WHO guidelines group. The WHO guidelines group is made up of members from diverse backgrounds, including users of services, which means that there is direct patient and public involvement (PPI) from the onset of this work. Our systematic review team also consists of people from diverse backgrounds and includes people who are current service users, as well as service users in the past, again indicating that PPI is integrated throughout this research and in the development of the research question. This diversity of backgrounds involved in our team is one of the strengths and reason why the WHO was keen to have our team complete this systematic review.

The Human Milk Banking Foundation (the guarantor of our WHO sponsored systematic review) is a patient organisation entered into the Polish register of the Patient Rights Ombudsman (RzPP-DWS.072.41.2023) representing the interests and needs of premature and sick children and their caregivers in the field of equal access to breast milk and the principles of financing nutritional therapy as part of the guaranteed benefit. The protocol consulted and will consult on an ongoing basis with representatives of the PPI organisation, including AW, representing the HMB Foundation as the WHO’s contractor.

## supplementary material

10.1136/bmjopen-2024-087653 online supplemental file 1

10.1136/bmjopen-2024-087653 online supplemental file 2

10.1136/bmjopen-2024-087653 online supplemental file 3
