# Supplementary material for: Assessing the impact of different donor milk treatments on infant health and growth: a systematic review protocol
Source: BMJ Open. 2024 Dec 27;14(12):e087653. doi: 10.1136/bmjopen-2024-087653 (PMC11683976; doi:10.1136/bmjopen-2024-087653)
Supplement: online supplemental file 2 [file bmjopen-14-12-s002.docx]

**Online Supplementary file 3**

**Preliminary data extraction template**

Data extraction sheet

**Study details**

- Study ID

- Reference

- Contact author details

- Country study was conducted in

- Study setting/s

- Trial registration number

- Sponsorship/funding source

- Other

**Study methods**

- Study aim/objective

- Study design

- Method of recruitment

- Timepoints and duration of follow-up

- Blinding

- Other

**Study population**

- Total sample size

- Inclusion criteria

- Exclusion criteria

- Withdrawal from study (number, reason, timing)

- Baseline characteristics (gestational age at birth, anthropometric data, morbidities)

- Group differences at baseline

- Other

**Study interventions**

- Indication of intervention (e.g. prematurity, post-surgery, formula intolerance)

- Intervention/comparison

- Number allocated to intervention/comparison group

- Duration of the intervention

- Amount of intervention received

- Setting of intervention (e.g. NICU, maternity ward, non hospitalised infants)

- Duration of follow-up

- Other

**Study outcomes**

- Outcome

- Type of variable, default measures

- Timepoints

- Scale of outcome measurement, range of the scale, direction

- Unit of measurement

**Other**
