# Supplementary material for: Assessing the impact of different donor milk treatments on infant health and growth: a systematic review protocol
Source: BMJ Open. 2024 Dec 27;14(12):e087653. doi: 10.1136/bmjopen-2024-087653 (PMC11683976; doi:10.1136/bmjopen-2024-087653)
Supplement: Supplementary file 4 [file bmjopen-2024-087653.reviewer_comments.pdf]

PEER REVIEW HISTORY

BMJ Open publishes all reviews undertaken for accepted manuscripts. Reviewers are asked to complete a checklist review form and are provided with free text boxes to elaborate on their assessment. These free text comments are reproduced below.

ARTICLE DETAILS

Title (Provisional)

Assessing the impact of different donor milk treatments on infant health and growth: a systematic review protocol

Authors

Klotz, Daniel; Bzikowska-Jura, Agnieszka; Giribaldi, Marzia; Cassidy, Tanya; Cavallarin, Laura; Gandino, Serena; Karcz, Karolina; Peila, Chiara; Smith, Carolyn; Walczak, Bartłomiej; Wesolowska, Aleksandra

---

VERSION 1 - REVIEW

**Reviewer**	**1**
**Name**	**Williams , Thomas**
**Affiliation**	**Simpson Centre for Reproductive Health**
**Date**	**14-May-2024**

COI **I am Principal Investigator for the BronchStart project, which is funded by the Respiratory Syncytial Virus Consortium in Europe (RESCEU), with data collection supported by the National Institute for Health Research.**

Globally, as childhood mortality rates fall, preterm birth and its complications are becoming the most common cause of mortality and morbidity in children. Therefore any study which seeks to assess and standardise the use of an intervention such as Donor Human Milk (DHM) is to be welcomed, particularly given the heterogeneity in practice regarding the donation, storage and administration of this milk. I am apprehensive that the authors of this systematic review may find insufficient evidence to support or refute particular practices related to the use of DHM, but I commend them on the drafting of a thorough, logical and systematic protocol.

My main comment is that, for a field where parent/carer involvement is known to have such a significant effect on outcomes, it seems a shame to have no formal PPI. I would urge the authors at the very least to consider sharing their protocol with a patient group so that members of this group could pick out factors which they felt to be of particular significance

in this patient population, and ensure that data on all outcomes judged to be important are gathered.

I also have a number of minor comments which may help to improve the clarity of the manuscript, listed below.

Minor comments:

The instructions to the reviewers state that "the dates of the study should be included in the manuscript". I have read through the manuscript but am unable to identify any dates apart from the 5 November deadline for the WHO submission. I would suggest explicitly adding a date for when the review is due to the start in a revised version of this manuscript.

Introduction, Line 106: The authors give the term NEC without defining what this is an abbreviation form. On this note, one of the main outcome measures in DHM trials has been necrotising enterocolitis, but this is not explicitly listed as an outcome in Table 2 or Supplementary File 3. I suggest that this be added as an outcome, alongside sepsis/suspected sepsis, and perhaps subdivided further into medically and surgically managed NEC. Furthermore, I suggest that in Table 2 growth should be expanded to encompass not just weight gain but other variables such as OFC; ideally these parameters should be expressed in a standardised measure such as Z-scores, otherwise comparisons of studies between different populations is likely to prove challenging.

Introduction, Line 122: "One of the crucial steps NEEDED to implement HMB worldwide".

Methods, Line 176: The following statement could be made clearer: "The reference list of relevant reviews will be screened manually to identify potentially additional relevant studies. We will hand search PubMed and Google Scholar for reference lists, cited by and similar articles (Higgins et al., 2023). This part of the search will be restricted to the 10 most cited articles we find in the main search and the first 200 records found in this way."

Do the authors mean the 10 most cited articles included in the references of an included study? And the first 200 records from what list, generated in which way? I am sure the authors know what they mean but they should communicate this more clearly to the non-expert reader.

Methods, Line 189 : The authors should briefly explain what the COVIDENCE system is, and cite it here, rather than later on, on line 197.

Methods, line 237: "using the Cochran Q and creating a forest plot". The authors should briefly explain what the Cochran Q test is, reference this, and explain what software they will use to perform the test.

Table 1: Intervention 5 and 6 appear to be the same.

**Reviewer**	**2**
**Name**	**Cacho , Nicole**
**Affiliation**	**University of California Davis, Pediatrics**
**Date**	**28-May-2024**
**COI**	**None**

Author Daniel Klotz and colleagues outline a planned systematic review on Donor Human Milk obtained from milk banks in their manuscript titled "The impact of feeding milk from donors with varying lactation stages, health concerns, varying expression practices or techniques, or with various treatment or testing methods on health and growth outcomes of infants fed donor milk - A systematic review protocol".

The authors highlighted the importance of creating a protocol to be used for future systematic review. There are changes on the horizon such as the WHO DHM guidelines that will generate further knowledge and highlight gaps in the literature. Utilization of this protocol will hopefully facilitate future global collaboration between the human milk banks.

This novel protocol is important to the field of pediatrics and neonatology because future systemic review findings may inform outdated practices of processing DHM and may delineate favorable desired outcomes in the most vulnerable infants. Donor human milk is widely used and well-designed systematic reviews are a necessary addition to the literature.

Major revision points include defining the PICOS points from Table 1.

-The study population is the most important part of the PICOS and when discussing DHM populations vary

In PICO, the population of infants receiving milk needs to be better defined. As the majority of donor human milk is prioritized for very low birth weight (VLBW) infants or critically ill infants, many studies will pertain to the population of VLBW infants. Other critically ill infants may include those with congenital heart disease, abdominal surgical issues, SGA or IUGR infants. Other populations who may receive donor human milk include late preterm infants or term infants in the newborn nursery for a short period of time. Although less common, there is a subset of neonates and infants who qualify to receive donor human milk in the outpatient setting. Therefore, the population of recipients of donor human milk must be better defined.

Outcomes will depend on your population. VLBWs have prolonged hospital stays and their growth (weight, height, head circumference and z-scores) are closely monitored. This may be different from infants with congenital heart disease who may initially have shorter hospital stays and are readmitted to the hospital for planned/staged surgeries.

If looking at the Newborn nursery population that may receive DHM, following growth during the 24-72 hour period of hospitalization would not be helpful since weight loss during the first week of life is expected in a neonate.

-Intervention 1 Lactation stage definition: Colostrum, transitional milk and mature milk or actual post-partum days? How will you define the lactation stage when there are multiple donors who contribute milk expressed on different days, and hence possibly different lactation stages?

-Intervention 2 Which birth outcomes? Term vs. Preterm? Or birthweight? Or more along the lines of congenital anomalies and/or genetic diagnosis?

-Intervention 4 Are lifestyle factors to this level of detail typically collected? My understanding is these are used as screening tools for eligibility for the donor. For instance, lactating parents who smoke are not eligible to be HMBANA donors.

-Intervention 5 and 6 are the same, please remove one.

-Intervention 9. Please provide some examples of different testing procedures? Is this Nutrient analysis or more along the lines of bacterial culture?

Additional Major revision points include defining health, growth and other outcomes from Table 2

-Morbidity: This is a large category and by better defining your population, you can better define outcomes. For instance if your population includes VLBWs, looking at NEC, ROP, Late onset sepsis, BPD would all be appropriate. When looking at a term infant in the newborn nursery, exclusive human milk and partial human milk intake as well as breastfeeding at discharge and subsequent, 2, 3, 6 and 12 month pediatrician appointments would be more appropriate.

-Mortality: Will you look at neonatal, or infant mortality? Or mortality up to a certain age?

-Tolerance: Does this refer to feeding tolerance/intolerance in the NICU? If so, state that specifically.

-Adverse effects/effects: What does this mean? This seems very vague

-Infections: Which infections, in which context over which time period? If looking at VLBW infants Late onset sepsis is appropriate. If looking at term infants perhaps Otitis media or diarrheal infections would be of interest.

-Nutritional deficiencies: What is meant by this? Vitamin deficiencies? How would this be measured? Again, what time period? Is this inpatient in VLBWs or outpatient in term health infants, or a combination of both?

Minor revision point

Clarify line 140-143: will you categorize which infants had which combinations of milk types and which proportions if available? Dose of human milk is an predictor of outcomes such as NEC and late onset sepsis in preterm infants.

VERSION 1 - AUTHOR RESPONSE

Reviewer: 1

Globally, as childhood mortality rates fall, preterm birth and its complications are becoming the most common cause of mortality and morbidity in children. Therefore any study which seeks to assess and standardise the use of an intervention such as Donor Human Milk (DHM) is to be welcomed, particularly given the heterogeneity in practice regarding the donation, storage and administration of this milk. I am apprehensive that the authors of this systematic review may find insufficient evidence to support or refute particular practices related to the use of DHM, but I commend them on the drafting of a thorough, logical and systematic protocol.

My main comment is that, for a field where parent/carer involvement is known to have such a significant effect on outcomes, it seems a shame to have no formal PPI. I would urge the authors at the very least to consider sharing their protocol with a patient group so that members of this group could pick out factors which they felt to be of particular significance in this patient population, and ensure that data on all outcomes judged to be important are gathered.

I also have a number of minor comments which may help to improve the clarity of the manuscript, listed below.

Dear Reviewer,

Thank you for your valuable feedback on our systematic review protocol. We agree that the heterogeneity in practices surrounding the donation, storage, and administration of DHM highlights the need for rigorous research to establish standardized guidelines. While we anticipate encountering challenges in synthesizing sufficient evidence to definitively support or refute specific DHM practices, our protocol was designed to systematically evaluate the available literature and contribute to a more informed understanding of this critical intervention.

We would like to thank You for Your observation regarding the need for a PPI. We have carefully addressed this point and included a specific statement addressing the issue.

Comments to the Authors:

Minor comments:

- The instructions to the reviewers state that "the dates of the study should be included in the manuscript". I have read through the manuscript but am unable to identify any dates apart from the 5 November deadline for the WHO submission. I would suggest explicitly adding a date for when the review is due to the start in a revised version of this manuscript.

Response: we have included the planned search dates in our protocol (line 177). These searches were conducted after the protocol submission and have provided the data foundation for our ongoing systematic review.

- Introduction, Line 106: The authors give the term NEC without defining what this is an abbreviation form. On this note, one of the main outcome measures in DHM trials has been necrotising enterocolitis, but this is not explicitly listed as an outcome in Table 2 or Supplementary File 3. I suggest that this be added as an outcome, alongside sepsis/suspected sepsis, and perhaps subdivided further into medically and surgically managed NEC.

Response: We tried to be as broad as possible in the present protocol, in order not to restrict too much the list of the possible articles retrieved. In the new Supplementary file on search strategy, the readers might find the terms that were planned to be searched for. These include "Outcome* OR impact* OR affect* OR influenc* OR result* OR consequenc* OR caus* OR issue* OR threat* OR effect* OR benefit* OR harm* OR "risk factor*" OR tolera* OR growth* OR development* OR neurodevelop* OR behavio* OR weight OR digest* OR diet OR nutrition OR malnutrition OR deficien* OR illness* OR death* OR morbidity OR mortality OR infection* OR transmi* OR sepsis* OR enterocolitis* OR cytomegalovirus* OR HIV OR immunologic OR diarrh* OR meningitis OR "urinary tract infection*" OR UTI OR Expos* OR Quality OR Complication*". We therefore plan to include specific analyses for the outcomes listed by the Reviewer in the final draft of the review. These are now better specified in Table 2 as well.

- Furthermore, I suggest that in Table 2 growth should be expanded to encompass not just weight gain but other variables such as OFC; ideally these parameters should be expressed in a standardised measure such as Z-scores, otherwise comparisons of studies between different populations is likely to prove challenging.

Response: We fully concur with this observation, and plan to include any growth measure, not only weight gain. Also, standardized measures will be searched for, although we might experience some limitation with older studies. To clarify this issue to the reader we amended the definition of outcome criteria which now reads (Table 1 an Table 2), and added to the main text (line 221-224): "We aim to incorporate any reported variations of health and growth outcome parameters to ensure inclusivity across all relevant outcome variables."

- Introduction, Line 122: "One of the crucial steps NEEDED to implement HMB worldwide".

Response: modified as suggested (line 117)

- Methods, Line 176: The following statement could be made clearer: "The reference list of relevant reviews will be screened manually to identify potentially additional relevant studies. We will hand search PubMed and Google Scholar for reference lists, cited by and similar articles (Higgins et al., 2023). This part of the search will be restricted to the 10 most cited articles we find in the main search and the first 200 records found in this way." Do the authors mean the 10 most cited articles included in the references of an included study? And the first 200 records from what list, generated in which way? I am sure the authors know what they mean but they should communicate this more clearly to the non-expert reader.

Response: we have carefully revised the statement to enhance its clarity and comprehensibility. The new formulation (lines 171-176) is now: "the search strategy will consist of search words on the key concepts of "donated human milk" and "effect on infants" (Online supplemental file 1). These search terms will be adapted for use with the other bibliographic databases. The 10 most cited articles among those retrieved by database search and included in the systematic review will be manually searched for in PubMed and Google Scholar for reference lists, cited by and similar articles [11]. The first 200 records for each of these articles found in this way in each database will undergo the same screening process as the parent article. Additionally, the reference list of relevant reviews will be screened manually to identify potentially additional relevant studies."

- Methods, Line 189 : The authors should briefly explain what the COVIDENCE system is, and cite it here, rather than later on, on line 197.

Response: We included the suggested explanation (lines 186-188).

Methods, line 237: "using the Cochran Q and creating a forest plot". The authors should briefly explain what the Cochran Q test is, reference this, and explain what software they will use to perform the test.

Response: We included the suggested explanation (lines 238-241).

- Table 1: Intervention 5 and 6 appear to be the same.

Response: We thank the reviewer to point out this error that we corrected. Intervention 6 now reads as: "when donated milk is expressed with different methods".

Reviewer: 2

Comments to the Author: Author Daniel Klotz and colleagues outline a planned systematic review on Donor Human Milk obtained from milk banks in their manuscript titled "The impact of feeding milk from donors with varying lactation stages, health concerns, varying expression practices or techniques, or with various treatment or testing methods on health and growth outcomes of infants fed donor milk - A systematic review protocol".

The authors highlighted the importance of creating a protocol to be used for future systematic review. There are changes on the horizon such as the WHO DHM guidelines that will generate further knowledge and highlight gaps in the literature. Utilization of this protocol will hopefully facilitate future global collaboration between the human milk banks.

This novel protocol is important to the field of pediatrics and neonatology because future systemic review findings may inform outdated practices of processing DHM and may delineate favorable desired outcomes in the most vulnerable infants. Donor human milk is widely used and well-designed systematic reviews are a necessary addition to the literature.

Dear Reviewer,

Thank you for your thoughtful comments on our protocol. We appreciate your recognition of the importance of conducting a systematic review to address the impact of various donor milk characteristics on infant health and growth outcomes. As you mentioned, the evolving guidelines from the World Health Organization (WHO) and the increasing use of donor human milk (DHM) make this research particularly timely. By examining the available literature, we hope to identify best practices for processing DHM and ultimately improve the outcomes for infants receiving this valuable nutritional resource.

Comments to the Authors:

Major revision points include defining the PICOS points from Table 1.

- The study population is the most important part of the PICOS and when discussing DHM populations vary. In PICO, the population of infants receiving milk needs to be better defined. As the majority of donor human milk is prioritized for very low birth weight (VLBW) infants or critically ill infants, many studies will pertain to the population of VLBW infants. Other critically ill infants may include those with congenital heart disease, abdominal surgical issues, SGA or IUGR infants. Other populations who may receive donor human milk include late preterm infants or term infants in the newborn nursery for a short period of time. Although less common, there is a subset of neonates and infants who qualify to receive donor human milk in the outpatient setting. Therefore, the population of recipients of donor human milk must be better defined. Outcomes will depend on your population.

Response: Based on the research question formulated by the WHO, we aimed for a comprehensive approach and included all relevant infant populations within the first year of life. Consequently, our search strategy focused on identifying "infant" populations without

imposing any restrictions on specific population definitions. This allowed for a broad and inclusive search scope.

To clarify this issue for the reader we added to the abstract (line 59): "No setting, patient population, date or language restrictions will be applied". We also added to the main text in line 146: We will not apply setting, patient population or time frame restrictions.

- VLBWs have prolonged hospital stays and their growth (weight, height, head circumference and z-scores) are closely monitored. This may be different from infants with congenital heart disease who may initially have shorter hospital stays and are readmitted to the hospital for planned/staged surgeries. If looking at the Newborn nursery population that may receive DHM, following growth during the 24-72 hour period of hospitalization would not be helpful since weight loss during the first week of life is expected in a neonate.

Response: We absolutely agree with the reviewer. As mentioned above, we tried to be as inclusive as possible and included any growth outcomes as reported by the included studies.

- Intervention 1 Lactation stage definition: Colostrum, transitional milk and mature milk or actual post-partum days? How will you define the lactation stage when there are multiple donors who contribute milk expressed on different days, and hence possibly different lactation stages?

Response: We agree with the reviewer that accurately defining the lactation stage is crucial when planning and conducting an interventional clinical trial involving human milk. To address this, we have designed our data extraction tool to capture the most commonly reported classifications of human milk as described in the studies we will review. However, other categorizations, such as pooled milk from different lactation stages, will require careful, additional consideration and reporting in the review.

- Intervention 2 Which birth outcomes? Term vs. Preterm? Or birthweight? Or more along the lines of congenital anomalies and/or genetic diagnosis?

Response: We agree with the reviewer that this phrasing may cause confusion. Therefore, we changed the phrasing as follows (Table 1): "Intervention 2 when donors are characterised by different birth outcomes (i.e. premature versus delivery at term) and added in line 114-115: "(i.e. premature vs term delivery)". We hope that this clarifies the issue for the reader.

- Intervention 4 Are lifestyle factors to this level of detail typically collected? My understanding is these are used as screening tools for eligibility for the donor. For instance, lactating parents who smoke are not eligible to be HMBANA donors.

Response: The reviewer raises a valid point. However, in alignment with the WHO's objectives, our goal was to specifically gather literature where any of these lifestyle factors were used as an intervention or a defining characteristic of a donor population, compared to donors without that lifestyle factor. While comparing smoking to non-smoking donors may be unrealistic, we included such comparisons and others, more realistic, e.g. such as vegan versus omnivore donors. The overarching aim of the review was to be as inclusive as possible in this regard.

- Intervention 5 and 6 are the same, please remove one.

Response: We thank the reviewer to point out this error that we corrected. Intervention 6 now reads as: "when donated milk is expressed with different methods".

- Intervention 9. Please provide some examples of different testing procedures? Is this Nutrient analysis or more along the lines of bacterial culture?

Response: We added clarifying information to Intervention which now reads: "when different testing procedures are used on donations (i.e. for microbial contamination)"

Additional Major revision points include defining health, growth and other outcomes from Table 2

- Morbidity: This is a large category and by better defining your population, you can better define outcomes. For instance if your population includes VLBWs, looking at NEC, ROP, Late onset sepsis, BPD would all be appropriate. When looking at a term infant in the newborn nursery, exclusive human milk and partial human milk intake as well as breastfeeding at discharge and subsequent, 2, 3, 6 and 12 month pediatrician appointments would be more appropriate.

- Mortality: Will you look at neonatal, or infant mortality? Or mortality up to a certain age?

- Tolerance: Does this refer to feeding tolerance/intolerance in the NICU? If so, state that specifically.

- Adverse effects/effects: What does this mean? This seems very vague

- Infections: Which infections, in which context over which time period? If looking at VLBW infants Late onset sepsis is appropriate. If looking at term infants perhaps Otitis media or diarrheal infections would be of interest.

- Nutritional deficiencies: What is meant by this? Vitamin deficiencies? How would this be measured? Again, what time period? Is this inpatient in VLBWs or outpatient in term health infants, or a combination of both?

Response: We appreciate these detailed analysis of the research questions and would like to address the aforementioned points with a combined response.

The primary and overarching aim of the WHO Guidelines group, which formulated these research questions, was to ensure inclusivity across all relevant factors. Consequently, we will undertake a comprehensive literature search that incorporates all the variables you mentioned. This broad approach is clearly outlined in our search strategy, as detailed in Online Supplementary File 2.

In executing this strategy, we will carefully analyze and extract data from eligible studies, paying particular attention to the concerns you raised. This will enable us to adequately address these issues in the review, ensuring that the final analysis thoroughly reflects the variables highlighted in your comments. We are committed to providing a well-rounded and inclusive response to the research questions.

To clarify this issue to the reader we amended the definition of outcome criteria which now reads (Table 1 an Table 2): Outcome: differently affected in their growth, development and/or health status (any reported outcome). We also added to the main text (line 221-224): "We aim to incorporate any reported variations of health and growth outcome parameters to ensure inclusivity across all relevant outcome variables."

- *Clarify line 140-143: will you categorize which infants had which combinations of milk types and which proportions if available? Dose of human milk is an predictor of outcomes such as NEC and late onset sepsis in preterm infants.*

Response: We agree with the reviewer, we aim to report combinations and proportions of milk feedings as both factors may severely impact findings and conclusions from clinical trials. Since we expect a large number of studies with very diverse study designs we subsumed the extraction of data like these as "Study intervention – intervention/comparison" in the data extraction template (Online supplementary file 3).

VERSION 2 - AUTHOR RESPONSE

Point by point response:

Major revision points include defining the PICOS points from Table 1.

- **The study population is the most important part of the PICOS and when discussing DHM populations vary. In PICO, the population of infants receiving milk needs to be better defined. As the majority of donor human milk is prioritized for very low birth weight (VLBW) infants or critically ill infants, many studies will pertain to the population of VLBW infants. Other critically ill**

infants may include those with congenital heart disease, abdominal surgical issues, SGA or IUGR infants. Other populations who may receive donor human milk include late preterm infants or term infants in the newborn nursery for a short period of time. Although less common, there is a subset of neonates and infants who qualify to receive donor human milk in the outpatient setting. Therefore, the population of recipients of donor human milk must be better defined. Outcomes will depend on your population. VLBWs have prolonged hospital stays and their growth (weight, height, head circumference and z-scores) are closely monitored. This may be different from infants with congenital heart disease who may initially have shorter hospital stays and are readmitted to the hospital for planned/staged surgeries. If looking at the Newborn nursery population that may receive DHM, following growth during the 24-72 hour period of hospitalization would not be helpful since weight loss during the first week of life is expected in a neonate.
The statement is now clarified in Introduction.

- **Intervention 1 Lactation stage definition: Colostrum, transitional milk and mature milk or actual post-partum days? How will you define the lactation stage when there are multiple donors who contribute milk expressed on different days, and hence possibly different lactation stages?**
We will follow the definition of the authors of the single studies: we will consider studies comparing "different lactation stages of the donors", so we might anticipate that different studies could include different definitions of these stages as post-partum days; we will report for such differences in definition when observed. If studies will compare milk from donors in a specific lactation stage (e.g: translational milk) with pooled composite milk, we will address these studies separately. Since the overall approach of the present systematic review protocol is to be as inclusive as possible, similar situations are not pre-defined, and, coherently, we do not include any specified "intervention" in the search strategy.

Additional Major revision points include defining health, growth and other outcomes from Table 2

Morbidity: This is a large category and by better defining your population, you can better define outcomes. For instance if your population includes VLBWs, looking at NEC, ROP, Late onset sepsis, BPD would all be appropriate. When looking at a term infant in the newborn nursery, exclusive human milk and partial human milk intake as well as breastfeeding at discharge and subsequent, 2, 3, 6 and 12 month pediatrician appointments would be more appropriate.
The statement is now further clarified in Outcome section of Methods.

- **Mortality: Will you look at neonatal, or infant mortality? Or mortality up to a certain age?**
We do not prespecified this criteria since we will include all studies reporting on mortality, irrespectively of the age. We included this specification in Table 2

- **Tolerance: Does this refer to feeding tolerance/intolerance in the NICU? If so, state that specifically.**
  We included this specification in Table 2
- **Adverse effects/effects: What does this mean? This seems very vague**
  We slightly modified the definition in Table 2 for Adverse effects to Adverse Events (any unwanted medical occurrence in a patient undergoing an intervention regardless of whether it is related to the intervention or not), and added a citation to one article reporting some examples of DHM related adverse events in both donor and recipients.
- **Infections: Which infections, in which context over which time period? If looking at VLBW infants Late onset sepsis is appropriate. If looking at term infants perhaps Otitis media or diarrheal infections would be of interest.**
  We do not prespecify any criteria since we will include all studies reporting on relevant infections, depending on the considered experimental setting, without restricting to specific pathogens or agents. We included this specification in Table 2
- **Nutritional deficiencies: What is meant by this? Vitamin deficiencies? How would this be measured? Again, what time period? Is this inpatient in VLBWs or outpatient in term health infants, or a combination of both?**
  We do not prespecified this criteria since we will include all studies reporting on nutrient deficiencies (either vitamins or micronutrient). We included this specification in Table 2
